# You Are Wanted

**Published:** 1861-10

**Authors:** 


### YOU ARE WANTED.

In the great battle between light and darkness, between truth and falsehood, between sin and holiness, every human being bears his part; is for, or against. There is no neutral position in that war. To do nothing, is to be against; and to be against the right, is to be lost. Idleness is a crime; indifference, a fatuity. There is much to do, and little time to do it in; for, "The night cometh, when no man can work." Work while the day lasts; work hard, work well; these should be the resolves of all the friends of a true Christianity, some of whom can do a great deal—all can do something, little though it may be; yet, that little is essential to the completion of the great work; as in a magnificent engine, it might as well lack a driving-wheel, as the smallest pin or most diminutive screw. Every temptation resisted, every passion curbed, every lust mortified, every pure desire cherished, every good deed done, every kind word spoken, every benignant look, every cheering smile, every sympathetic throb for a brother's sorrow or a sister's tear, is something done towards the elevation of humanity to its high seat, hard by the Throne of God. And as there is not a human being but can do some of these things, there is work for all, and work that all can do. What magnificent encouragement is there, then, in the consciousness that the creature can become a co-worker with his Creator; a worm be made a fellow-laborer with the Omnipotent. That Omnipotence is the embodiment of Love, for "God is Love;" "his loving kindness is over all his works," and, most of all, over man, whose happiness here and hereafter, is an object of his care, to the extent of giving his only and well-beloved Son to become an adjudged culprit on the cross, that man thereby might be made immortally blessed.

If, then, humanly speaking, the Father of us all has made such sacrifices to promote the happiness of man, his child, and has put it in our power to engage with him in that work, securing eternal life as the wages for it, there is no nobler spectacle in the universe, than that of a man, every outgoing of whose heart is in loving kindness towards all of woman born, and in so doing, is learning here to assimilate himself to his Maker, coming nearer and nearer the pattern of the great Original every day, until life's latest hour, when he goes upward, to "be like Him," to "see Him as He is."

